# miR-29 as diagnostic biomarkers for tuberculosis: a systematic review and meta-analysis

**DOI:** 10.3389/fpubh.2024.1384510

**Published:** 2024-05-14

**Authors:** Jie He, Juan Xiong, Yuanyuan Huang

**Affiliations:** ^1^Clinical Medical College of Chengdu Medical College, Chengdu, Sichuan, China; ^2^Department of Pulmonary and Critical Care Medicine, The First Affiliated Hospital of Chengdu Medical College, Chengdu, Sichuan, China; ^3^Emergency Department, The First Affiliated Hospital of Chengdu Medical College, Chengdu, Sichuan, China

**Keywords:** tuberculosis, miR-29, diagnosis, biomarkers, meta-analysis

## Abstract

**Background:**

The timely diagnosis of tuberculosis through innovative biomarkers that do not rely on sputum samples is a primary focus for strategies aimed at eradicating tuberculosis. miR-29 is an important regulator of tuberculosis pathogenesis. Its differential expression pattern in healthy, latent, and active people who develop tuberculosis has revealed its potential as a biomarker in recent studies. Therefore, a systematic review and meta-analysis were performed for the role of miR-29 in the diagnosis of tuberculosis.

**Methods:**

EMBASE, PubMed, CNKI, Web of Science, and Cochrane Library databases were searched utilizing predefined keywords for literature published from 2000 to February 2024.Included in the analysis were studies reporting on the accuracy of miR-29 in the diagnosis of tuberculosis, while articles assessing other small RNAs were not considered. All types of study designs, including case–control, cross-sectional, and cohort studies, were included, whether prospectively or retrospectively sampled, and the quality of included studies was determined utilizing the QUADAS-2 tool. Publication bias was analyzed via the construction of funnel plots. Heterogeneity among studies and summary results for specificity, sensitivity, and diagnostic odds ratio (DOR) are depicted in forest plots.

**Results:**

A total of 227 studies were acquired from the various databases, and 18 articles were selected for quantitative analysis. These articles encompassed a total of 2,825 subjects, primarily sourced from the Asian region. Patient specimens, including sputum, peripheral blood mononuclear cells, cerebrospinal fluid and serum/plasma samples, were collected upon admission and during hospitalization for tuberculosis testing. miR-29a had an overall sensitivity of 82% (95% CI 77, 85%) and an overall specificity of 82% (95% CI 78, 86%) for detecting tuberculosis. DOR was 21 (95% CI 16–28), and the area under the curve was 0.89 (95% CI 0.86, 0.91). miR-29a had slightly different diagnostic efficacy in different specimens. miR-29a showed good performance in both the diagnosis of pulmonary tuberculosis and extrapulmonary tuberculosis. miR-29b and miR-29c also had a good performance in diagnosis of tuberculosis.

**Conclusion:**

As can be seen from the diagnostic performance of miR-29, miR-29 can be used as a potential biomarker for the rapid detection of tuberculosis.

**Systematic Review Registration:**

https://www.crd.york.ac.uk/PROSPERO/display_record.php?RecordID=461107.

## 1 Introduction

Tuberculosis (TB) diagnosis continues to pose a worldwide challenge, and misdiagnoses have contributed to heightened morbidity and mortality rates (1). Hence, the imperative lies in the identification of TB infection biomarkers to facilitate the diagnosis of active TB. Among the assessed TB biomarkers, microRNA (miRNA) is one such type under evaluation (2). miRNAs have been proposed as a novel biological diagnostic indicator in various settings encompassing diabetes, heart disease, infectious diseases, pregnancy, cancer, and psoriasis. *Mycobacterium tuberculosis* is among the numerous microorganisms that, upon infection, lead to modifications in epigenetic patterns within the host. Epigenetic processes have the capacity to modify gene expression without making changes to the underlying DNA sequence. These processes encompass DNA methylation, histone modifications, and the action of miRNAs (3). MiRNA stands as a promising avenue for application as a molecular diagnostic marker in diverse infectious diseases, serving as biomarkers for disease diagnosis, treatment outcome, and prognosis.

In addition, miRNA has been implicated in various diseases such as immunity, heart, infectious diseases, and cancer (4). As the disease progresses, the affected organs have the capacity to release specific miRNAs into the bloodstream. Compared to healthy individuals, miRNA levels in blood significantly differ in individuals with multiple diseases. Up until now, miRNAs have been utilized as molecular diagnostic indicators for a range of conditions, including cancer, mental disorders, diabetes, heart disease, and infectious diseases (5).

An essential element of the World Health Organization’s 2035 milestone strategy to eradicate TB involves the early diagnosis of the disease through systematic screening of high-risk populations. This call was made to identify and validate clear biomarkers capable of discriminating between active and latent TB, as well as predicting disease progression. Circulating miRNA has emerged as a potent biomarker in TB diagnosis (6). Furthermore, miRNA has been employed as a biological indicator to differentiate between latent and active TB (7) and as a biomarker for TB coinfection in individuals with HIV (8). Recent studies have also showcased the significant role of these biomarkers in predicting the progression of the disease (9). In prior investigations, microarray technology has been employed in multiple studies to pinpoint miRNAs as potential biomarkers for TB in multiple sample sources, such as serum, blood, sputum macrophages, and peripheral blood mononuclear cells of TB patients (10–12). For individuals with active pulmonary TB, previous studies identified 59 kinds of miRNAs upregulated and 33 kinds of miRNAs downregulated compared with healthy controls (10). Among the upregulated miRNAs, miRNA-29 family (miR-29a, miR-29b, and miR-29c), particularly miR-29a, stands out, as it inhibits immune responses by post-transcriptionally inhibiting interferon (INF)-γ expression in T cells. This mechanism potentially enhances susceptibility to TB (13). In the context of *Mycobacterium tuberculosis* infection, the miR-29 family assumes a crucial role in influencing innate and adaptive immunity by exerting an impact on gene expression within macrophages, dendritic cells, B cells, and T cells (14). Multiple *in vitro* and *in vivo* studies have highlighted the function of the miR-29 family in host immune regulatory mechanisms during TB pathogenesis. These mechanisms encompass the modulation of apoptotic pathways, induction of autophagy, stimulation of IFN-γ, and secretion of TNF-α. *Mycobacterium tuberculosis* uses a variety of strategies to counteract these regulatory mechanisms and enhance its survival capabilities (15–17).

In this research, the available literature on the potential of the miR-29 family as a diagnostic biomarker for tuberculosis was reviewed, and the reported diagnostic accuracy of miR-29 was evaluated. The candidate gene miR-29 was identified, which could be employed as a reliable diagnostic marker for TB.

## 2 Methods

The protocol has been registered in the PROSPERO registry (ID: CRD42023461107).^1^

### 2.1 Search strategy

The systematic assessment and subsequent meta-analysis followed the guidelines established by the Preferred Reporting Items for Systematic Reviews and Meta-Analyses (PRISMA) (18), and a corresponding report was generated. Extensive literature searches in the PubMed and CNKI databases were finished, and articles from the Web of Science and Embase databases were also retrieved. The search platform encompassed the inclusion of the Cochrane Library as well. MeSH vocabulary and keywords pertaining to TB and miR-29 were employed for the search, as outlined in the Supplementary Table 1. To identify potential additional eligible studies, both forward and backward citation checks were conducted based on relevant review articles.

### 2.2 Criteria for eligibility

#### 2.2.1 Time frame

Articles published in Chinese and English from January 2000 to February 2024 were encompassed in the study.

#### 2.2.2 Study type

All types of study designs, including case–control, cross-sectional, and cohort studies, were included, whether prospectively or retrospectively sampled. Non-original articles, such as narrative and systematic reviews, meta-analyses, and conference abstracts, were excluded. Animal studies were also not included.

#### 2.2.3 Biomarker criteria

Included in the analysis were studies reporting on miR-29, while articles assessing other small RNAs were not taken into account. In light of the defined scope of this systematic review, studies that had assessed miR-29 as a biological marker for diseases other than respiratory diseases and TB were excluded. Studies that lacked specificity, sensitivity, and area under the curve (AUC) data were not taken into consideration.

#### 2.2.4 Study population

No exclusion criteria were applied based on patient characteristics, encompassing studies involving both adult and pediatric populations.

### 2.3 Ethical review

Meta-analyses are a form of research that analyses previous research data without requiring ethical approval.

### 2.4 Screening and data extraction

All research articles identified through the search were imported into Endnote X9(EndNote X9, Clarivate Analytics, Philadelphia, PA, United States), and duplicate records were eliminated. The management of the included studies was conducted using Rayyan (19). Initially, two researchers conducted the screening of titles and abstracts from electronically retrieved publications to determine potentially eligible studies. Then, they subsequently conducted independent full-text screening to identify pertinent studies. In cases where specific articles lacked sufficient or complete data for a definitive inclusion or exclusion decision, the authors of the respective papers were contacted to obtain any absent data. The following data points were collected: publication year, sample size (cases and controls recorded separately), country, mean or median age, male-to-female ratio, study population (active TB/latent tuberculosis infection (LTBI)/healthy), sample source, reference test, screening and validation methods, identified miRNA (miR-29a, miR-29b, and miR-29c), and diagnostic accuracy indicators (specificity, sensitivity, and AUC). A data sheet was designed, and extracted data was entered into an Excel database. The values for True Positive (TP), False Positive (FP), True Negative (TN), and False Negative (FN) derived from the index test outcomes were documented. In cases where these values were not explicitly reported, they were computed based on the reported specificity, sensitivity, and sample size. For studies that did not contain adequate information to create a 2 × 2 table, the study authors were contacted and requested to provide the necessary details. If the study author could not provide this information, the study was not included in the meta-analysis, but the qualitative analysis within the narrative analysis section was retained. Two reviewers validated the extracted data, resolving any disagreements or discrepancies by discussion until a consensus was reached.

### 2.5 Evaluation of the quality of included studies

Methodological quality was evaluated for all-encompassed studies utilizing QUADAS-2 (Quality Assessment of Diagnostic Accuracy Studies) (20). The QUADAS-2 instrument includes four domains—patient selection, indicator testing, reference standards, and processes and timing, aimed at assessing the risk of bias and clinical applicability of the study. Within each domain, specific questions are posed to determine whether bias and applicability are rated as “low,” “high,” or “uncertain.” Studies were deemed to have good quality if they provided critical information, including details about biomarker identification methods and accurate classification of the study cohort (active or latent TB and control groups).

### 2.6 Data synthesis and statistical analysis

Meta-Disc 1.4(version 1.4; Universidad Complutense, Barcelona, Spain) and Stata 11.0(version 11.0; StataCorp LLC, College Station, TX, United States) software were utilized for all calculations. Since miR-29a is different from miR-29b and miR-2c, we separated them in our analyses. Spearman correlation coefficient was utilized to explore the presence of a threshold effect. Heterogeneity between studies was assessed utilizing the I^2^ test with a critical point of ≥50% and a *p* value of <0.10. In cases where heterogeneity was observed, a random-effects model was applied. Conversely, a fixed-effects model was utilized when no significant heterogeneity was detected. Pooled statistics were presented by specificity, sensitivity, positive likelihood ratio (PLR), negative likelihood ratio (NLR), diagnostic odds ratio (DOR), and 95% confidence interval. A summary receiver operating characteristic curve (SROC curve) was plotted, and the area under the curve (AUC) was computed. Subgroup analysis was used to further investigate the diagnostic efficacy of miR-29(miR-29a, miR-29b, and miR-29c) in different subgroups. Furthermore, publication bias was determined by generating the funnel chart (Deeks’ funnel plot). In full-text statistics, *p* < 0.05 was deemed as a statistically significant value. Besides, the results of individual studies will be summarized descriptively if fewer studies are unable to carry out a meta-analysis.

## 3 Results

### 3.1 Attributes of included studies

**Figure 1:**
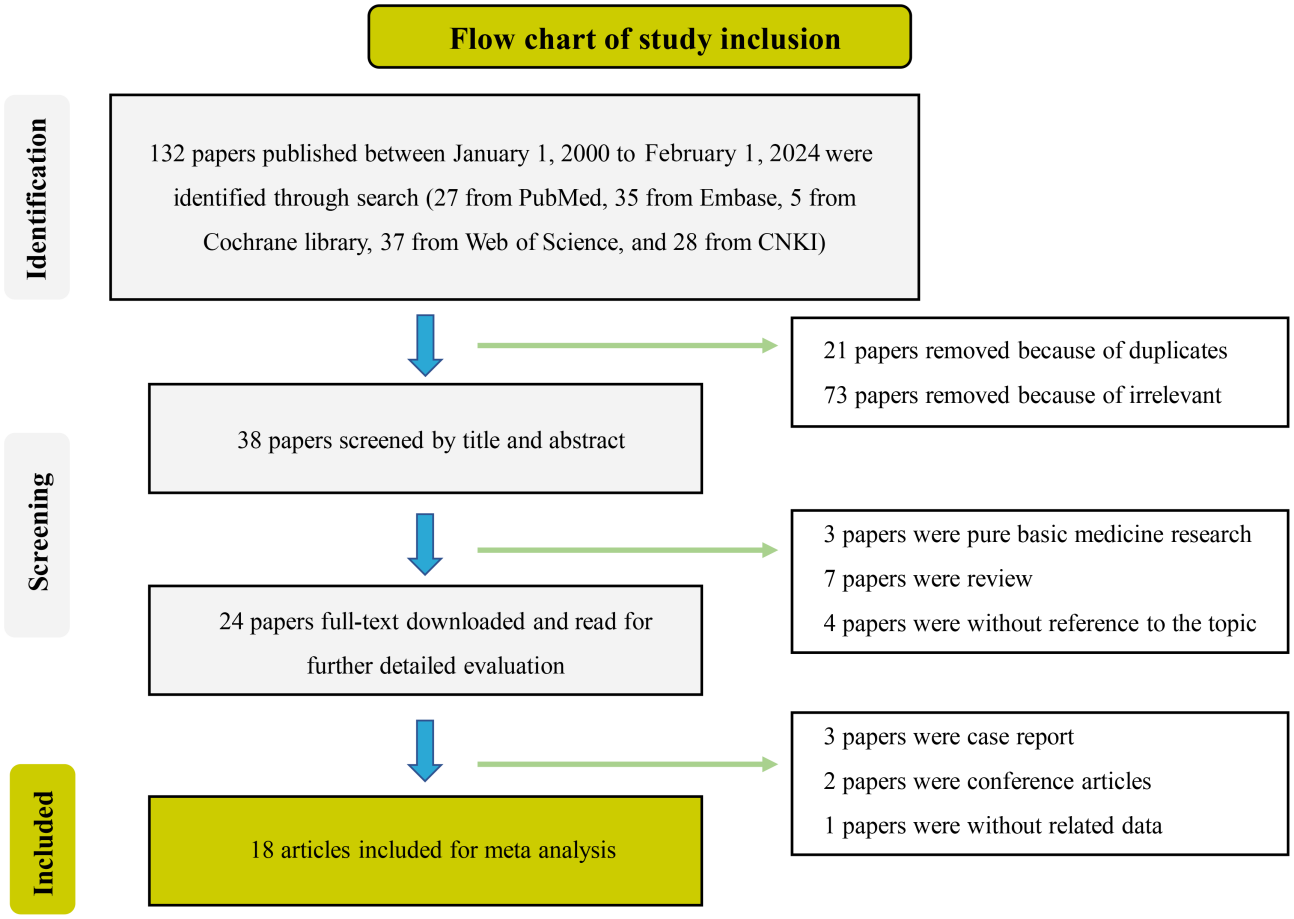
Detailed process of literature identification and screening.

**Figure 2:**
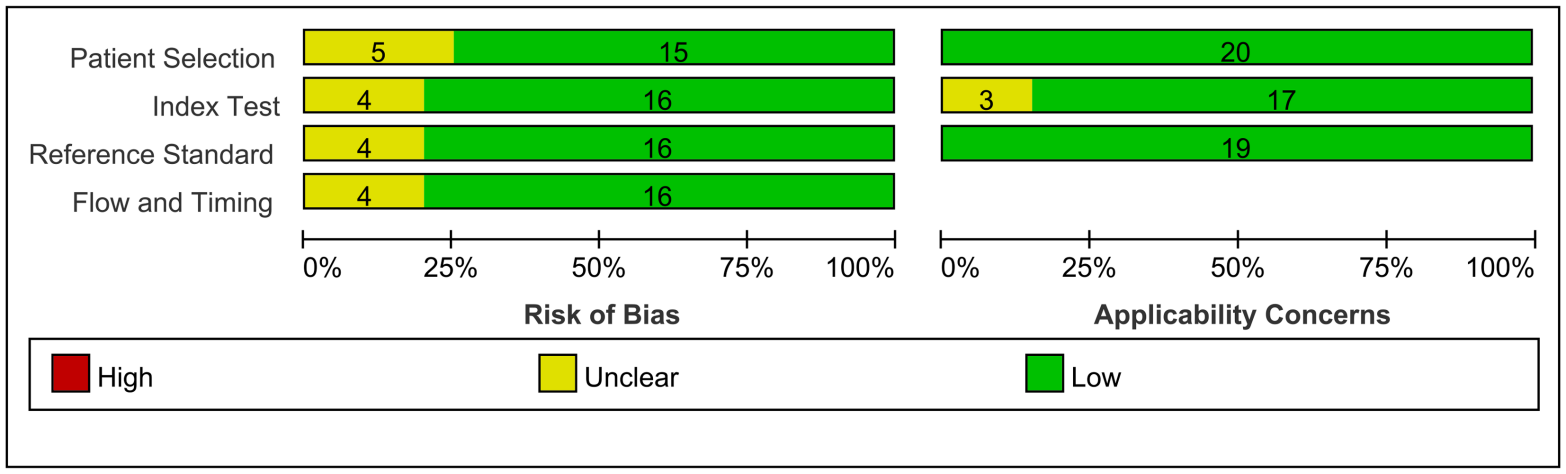
Overview of applicability concerns and the risk of bias of included studies as per the QUADAS-2 tool.

Out of the initial pool of 132 papers identified through the search formula, a total of 18 papers (3, 10, 11, 21–35) encompassing 20 studies were ultimately included in the analyses. This selection process involved the exclusion of irrelevant articles, duplicate studies, case reports, reviews, and other ineligible publications. These studies were mainly from Asian regions, involving 19 studies of active TB, 1 latent TB, 17 pulmonary TB, 3 extrapulmonary TB, 14 adult TB, and 3 pediatric TB. Eighteen studies on miR-29a, one on miR-29b, and one on miR-29c met inclusion criteria. Details of each included study are depicted in Tables 1, 2. The specific process of inclusion and exclusion is shown in Figure 1. Next, the quality assessment of the studies included in the analysis was performed using the QUADAS-2 tool. Studies that did not meet the inclusion criteria were assessed to have a high risk of bias (Figure 2).

**Table 1:** Characteristic of the included studies.

Authors	Year	Country	Patient characteristics						Diagnosis
			Control	Male/Female	Age (Years)	Cases	Male/Female	Age (Years)	
Yan BS	2016	China	98	51/47	39.7 ± 18.2	44	24/20	37.7 ± 18.5	Sputum smear microscopy, Culture, Radiological findings, T-SPOT, Pathological diagnosis.
Cai QS	2016	China	30	15/15	45.2 ± 6.9	30	18/12	44.7 ± 5.9	Clinical signs, Sputum smear microscopy, Culture, Pathological diagnosis, QFT-GIT
Chen XF	2017	China	45	NA	NA	45	NA	NA	Clinical signs, Radiological findings, Sputum smear microscopy, Culture
Liu YM	2015	China	60	36/24	49 ± 10.3	60	31/29	52.27 ± 13.89	Sputum smear microscopy, Culture, TB-DNA
Cao SL	2014	China	60	26/34	41.2 ± 15.6	40	20/20	43.2 ± 17.1	Clinical signs, Sputum smear microscopy, Culture, QFT
Ndzi E N	2019	India	42	13/29	27 ± 7	118	65/53	33 ± 12	Clinical signs, Sputum smear microscopy, Culture, Radiological findings, QFT
Wagh V	2017	China	30	26/4	28.71 ± 6.34	30	26/4	32.46 ± 12.03	Clinical signs, Radiological findings, Sputum smear microscopy, Culture
Pan D (Tuberculosis meningitis)	2017	China	130	68/62	1–8	122	65/57	1–8	Sputum smear microscopy, acid-fast bacilli (AFB) in cerebrospinal fluid (CSF) microscopy, Culture, Clinical signs of meningitis
Zhan J	2016	China	78	45/33	34.5 ± 21.3	151	93/58	32.5 ± 13.2	Clinical signs, Radiological findings, TGRA
Zhou M	2016	China	21	NA	NA	25	NA	7.5 ± 5.3	Clinical signs, Radiological findings, Sputum smear microscopy, Culture, NAAT, TST, IGRA
Yi ZJ	2012	China	28	15/13	NA	50	32/18	12–70	Clinical signs, Radiological findings, Sputum smear microscopy, Culture
Li Z	2020	China	186	101/85	45.63 ± 4.16	192	119/73	47.24 ± 5.38	Clinical signs, Radiological findings, Sputum smear microscopy, Culture
Angria N (Active tuberculosis)	2022	India	30	7/23	37.83 ± 15.74	50	34/16	41.04 ± 12.32	Clinical signs, Sputum smear microscopy, MGIT, IGRA, QFT
Angria N (Latent tuberculosis)	2022	India	30	7/24	37.83 ± 15.75	33	9/24	39.03 ± 12.66	Clinical signs, Sputum smear microscopy, MGIT, IGRA, QFT
Pedersen JL	2021	Australia	100	57/43	35(18–78)	100	58/42	43(19–91)	Clinical signs, Radiological findings, Sputum smear microscopy, Culture
Fu Y	2011	China	30	18/12	38.2 ± 17.3	30	21/9	41.2 ± 21.1	Clinical signs, Radiological findings, Sputum smear microscopy, Culture
Zhang X	2013	China	108	76/32	48(24–67)	128	86/42	41(30–64)	Clinical signs, Radiological findings, Sputum smear microscopy, Culture, TST
Zhang GD	2021	China	45	23/22	40.31 ± 20.22	52	26/26	38.72 ± 16.13	Sputum smear microscopy, Culture, Radiological findings, TGRA, TST
Yin H (Epididymal tuberculosis)	2022	China	60	60/0	31.84 ± 8.19	60	60/0	32.25 ± 8.36	Pathological diagnosis, TB-DNA

TB, Tuberculosis; NA, Not available; MGIT, Mycobacteria growth indicator tube; TST, Tuberculin Skin Test; IGRA, Interferon Gamma Release Assay; QFT-GIT, QuantiFERON Gold-In-Tube; NAAT, Nucleic Acid Amplification Test.

**Table 2:** Data extracted from included studies to perform qualitative and quantitative analysis.

Authors	Sample used	MiRNA profile	Validation method	Cut off value	AUC	Sensitivity (%)	Specificity (%)
Yan BS	PBMC	MiR-29a	RT-PCR	NA	0.939	90.91	81.63
Cai QS	Plasma	MiR-29a	RT-PCR	NA	0.732	80.0	69.2
Chen XF	Serum	MiR-29a	RT-PCR	NA	0.874	66.7	93.3
Liu YM	PBMC	MiR-29a	RT-PCR	NA	0.714	93	85
Cao SL	Plasma	MiR-29a	RT-PCR	NA	0.687	67.6	75
Ndzi EN	Plasma	MiR-29a	RT-PCR	NA	0.81	80	71
Wagh V	Serum	MiR-29a	RT-PCR	NA	0.68	87	80
Pan D	PBMC	MiR-29a	RT-PCR	0.807	0.852	67.2	88.5
Pan D	CSF	MiR-29a	RT-PCR	1.187	0.890	81.1	90.0
Zhan J	Plasma	MiR-29a	RT-PCR	NA	NA	94	64
Zhou M	PBMC	MiR-29b	RT-PCR	2.026	0.697	56	90.5
Yi ZJ	Sputum	MiR-29a	RT-PCR	NA	0.867	80	85
Li Z	Serum	MiR-29a	RT-PCR	1.842	0.818	89.78	70.83
Angria N (Active tuberculosis)	Serum	MiR-29a	RT-PCR	0.01	0.76	86	73
Angria N (Latent tuberculosis)	Serum	MiR-29a	RT-PCR	0.01	0.808	84.8	70
Pedersen JL	Serum	MiR-29a	RT-PCR	NA	0.927	83.3	80
Fu Y	Serum	MiR-29a	RT-PCR	NA	0.831	83	80
Zhang X	Serum	MiR-29c	RT-PCR	NA	0.846	72.6	82.8
Zhang GD	Plasma	MiR-29a	RT-PCR	1.13	0.753	68.2	80.2
Yin H	Serum	MiR-29a	RT-PCR	0.81	0.908	81.67	88.33

CSF, Cerebrospinal fluid; PBMC, Peripheral blood mononuclear cells.

### 3.2 Literature data extraction

These 20 studies involved 2,543 participants. Each study clearly described the sensitivity and specificity of miR-29 for TB diagnosis. miR-29 was analyzed by quantitative PCR in all studies, but the sources of specimens varied, with sputum in one study, peripheral blood mononuclear cells in 4 studies, cerebrospinal fluid in one study, serum in nine studies, and plasma in 5 studies. Eight studies clearly provided the data of best cut-off values.

### 3.3 Meta-analysis

Of the included studies, 18 studies were on miR-29a, one reported miR-29b, and one reported miR-29c, thus, diagnostic meta-analysis was performed on miR-29a.The remaining data (miR-29b and miR-29c) were analyzed descriptively.

#### 3.3.1 Threshold effect analysis

A threshold effect emerges due to variations in sensitivity, specificity, and DOR of the diagnostic test resulting from different thresholds. Therefore, it is essential to initially examine whether the diagnostic method exhibits a threshold effect. To assess this, Spearman correlation analysis was conducted utilizing Meta-Disc 1.4 software, yielding a correlation coefficient of *r* = 0.396 (*p* = 0.104). The absence of a significant threshold effect was indicated, allowing for the subsequent pooled analysis to proceed.

#### 3.3.2 Pooled analysis

**Figure 3:**
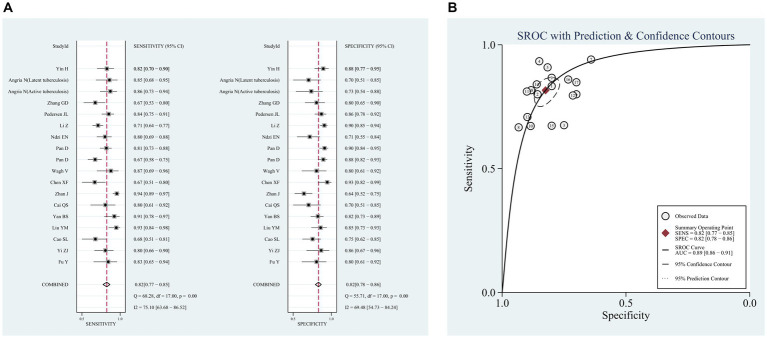
Pooled analysis of the diagnostic accuracy of miR-29a for patients with tuberculosis. **(A)** Sensitivity and Specificity, **(B)** SROC curve.

The pooled sensitivity and specificity across the 18 studies were 82% (95% CI, 77, 85%, I^2^ = 75.1%) and 82% (95% CI, 78, 86%, I^2^ = 69.5%), respectively (Figure 3A). The PLR was 4.7 (95% CI, 3.8, 5.7), and the NLR was 0.22 (95% CI, 0.18, 0.28). DOR was 21 (95% CI, 16, 28), and the AUC was 0.89 (95% CI, 0.86, 0.90; Table 3). The SROC curve of miR-29a with its confidence and prediction regions is shown in Figure 3B. In addition, the data on active TB was pooled for analysis to investigate the diagnostic ability of the miR-29a test for active TB. The diagnostic sensitivity and specificity for active TB were 81% (95% CI, 77, 85%) and 83% (95% CI, 79, 86%).

**Table 3:** Subgroup analysis of the included studies based on the differences of the purpose of testing and the positive result definition (miR-29a).

Subgroup(n)	Pooled sensitivity (95% CI)	I^2^	Pooled specificity (95% CI)	I^2^	Pooled positive likelihood ratio (95% CI)	I^2^	Pooled negative likelihood ratio (95% CI)	I^2^	Pooled diagnostic DOR (95% CI)	I^2^	AUC (95% CI)
Overall (18)	82% (77–85) 75.1%		82% (78–86) 69.5%		4.7 (3.8–5.7) 45.5%		0.22 (0.18–0.28) 66.9%		21 (16–28) 41.3%		0.89 (0.86–0.91)
Focal sites											
Pulmonary tuberculosis (15)	81% (78–83) 77.2%		81% (78–84) 65.3%		4.0 (3.2–4.9) 56.5%		0.24 (0.18–0.30) 65.9%		19 (13–26) 41.9%		0.88 (0.85–0.90)
Extrapulmonary tuberculosis (3)	76% (70–80) 74.3%		89% (85–92) 0.0%		6.9 (5.0–9.4) 0.0%		0.26 (0.17–0.41) 75.4		26 (14–48) 44.8%		0.96 (0.94–0.98)
Age											
Children (2)	74% (68–80) 84.0%		89% (85–93) 0.0%		6.8 (4.8–9.7) 0.0%		0.28 (0.16–0.50) 84.2%		24 (10–59) 69.1%		-
Adult (14)	82% (79–84) 76.7%		81% (78–83) 64.5%		3.9 (3.1–4.9) 58.4%		0.22 (0.17–0.29) 67.4%		19 (13–27) 47.7%		0.88 (0.85–0.91)
Sample source											
Serum (8)	81% (75–86) 56.9%		84% (79–89) 57.8%		5.2 (3.9–7.0) 0.0%		0.23 (0.17–0.30) 36.1%		23 (16–33) 0.0%		0.90 (0.87–0.92)
Plasma (5)	80% (68–89) 87.0%		72% (65–79) 2.8%		2.9 (2.4–3.6) 0.0%		0.27 (0.16–0.44) 77.5%		11 (6–19) 44.5%		0.79 (0.75–0.82)
PBMC (3)	79% (73–84) 91.4%		85% (81–89) 4.5%		5.5 (4.1–7.3) 0.0%		0.16 (0.04–0.55) 89.2%		34 (13–94) 67.8%		0.92 (0.88–0.95)

PBMC, Peripheral blood mononuclear cell.

#### 3.3.3 Subgroup analyses

##### 3.3.3.1 Lesion location of TB

**Figure 4:**
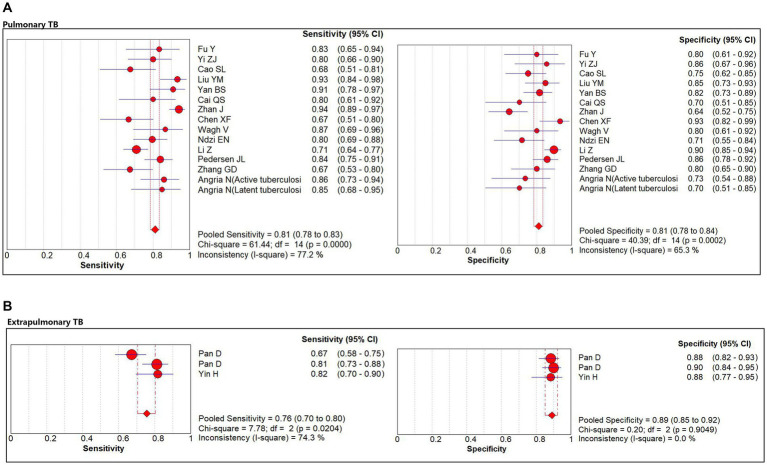
The sensitivity and specificity of miR-29a for patients with pulmonary TB and extrapulmonary TB. **(A)** Pulmonary TB. **(B)** Extrapulmonary TB. TB, tuberculosis.

The subgroup analysis was conducted according to the site of TB occurrence, aiming to further explore the origin of heterogeneity. Subgroup analysis showed that miR-29a was used for pulmonary TB screening with a sensitivity of 81% (95% CI: 78, 83%), specificity of 81% (95% CI: 78, 84%; Figure 4A), PLR of 4.0 (95% CI: 3.2, 4.9), NLR of 0.24 (95% CI: 0.18, 0.30), DOR of 19 (95% CI: 13, 26), and AUC of 0.88 (95% CI: 0.85, 0.90). When miR-29a was used for screening extrapulmonary TB, it had a sensitivity of 76% (95% CI: 70, 80%), specificity of 89% (95% CI: 85, 92%; Figure 4B), PLR of 6.9 (95% CI: 5.0, 9.4), NLR of 0.26 (95% CI: 0.17, 0.41), DOR of 26 (95% CI: 14, 48), and AUC of 0.96 (95% CI: 0.94, 0.98; Table 3).

##### 3.3.3.2 Age

The subgroup analysis was performed according to age. In the adult group, miR-29a had a sensitivity of 82% (95% CI: 79, 84%), specificity of 81% (95% CI: 78, 83%), PLR of 3.9 (95% CI: 3.1, 4.9), NLR of 0.22 (95% CI: 0.17, 0.29), DOR of 19 (95% CI: 13, 27), and AUC of 0.88 (95% CI: 0.85, 0.91) for the diagnosis of TB. In the pediatric group, miR-29a had a sensitivity of 74% (95% CI: 68, 80%), specificity of 89% (95% CI: 85, 93%), PLR of 6.8 (95% CI: 4.8, 9.7), NLR of 0.28 (95% CI: 0.25, 0.37), DOR of 24 (95% CI: 10, 59) for the diagnosis of TB (Table 3).

##### 3.3.3.3 Specimens

The subgroup analysis was executed according to the origin of the specimens. miR-29a in serum had a sensitivity of 81% (95% CI: 75, 86%), specificity of 84% (95% CI: 79, 89%), PLR of 5.2 (95% CI: 3.9, 7.0%), NLR of 0.23 (95% CI: 0.17, 0.30), DOR of 23 (95% CI: 16, 33), and AUC of 0.90 (95% CI: 0.87, 0.92) for the diagnosis of TB. miR-29a in plasma had a sensitivity of 80% (95% CI: 68, 89%), specificity of 72% (95% CI: 65, 79%), PLR of 2.9 (95% CI: 2.4, 3.6), NLR of 0.27 (95% CI: 0.16, 0.44), DOR of 11 (95% CI: 6, 19), and AUC of 0.79 (95% CI: 0.75, 0.82) for the diagnosis of TB. Furthermore, miR-29a in peripheral blood mononuclear cell plasma had a sensitivity of 79% (95% CI: 73, 84%), specificity of 85% (95% CI: 81, 89%), PLR of 5.5 (95% CI: 4.1, 7.3), NLR of 0.16 (95% CI: 0.04, 0.55), DOR of 34 (95% CI: 13, 94), and AUC of 0.92 (95% CI: 0.88, 0.95) for the diagnosis of TB (Table 3).

#### 3.3.4 Sensitivity analysis and publication bias analysis

**Figure 5:**
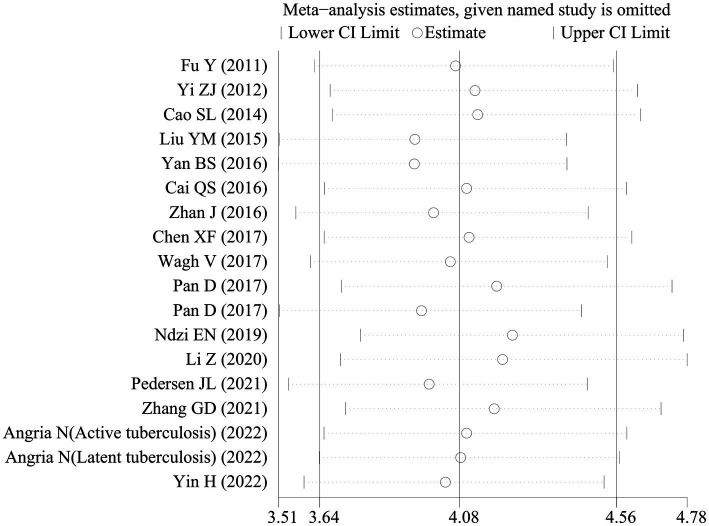
Sensitivity analysis of the included studies.

**Figure 6:**
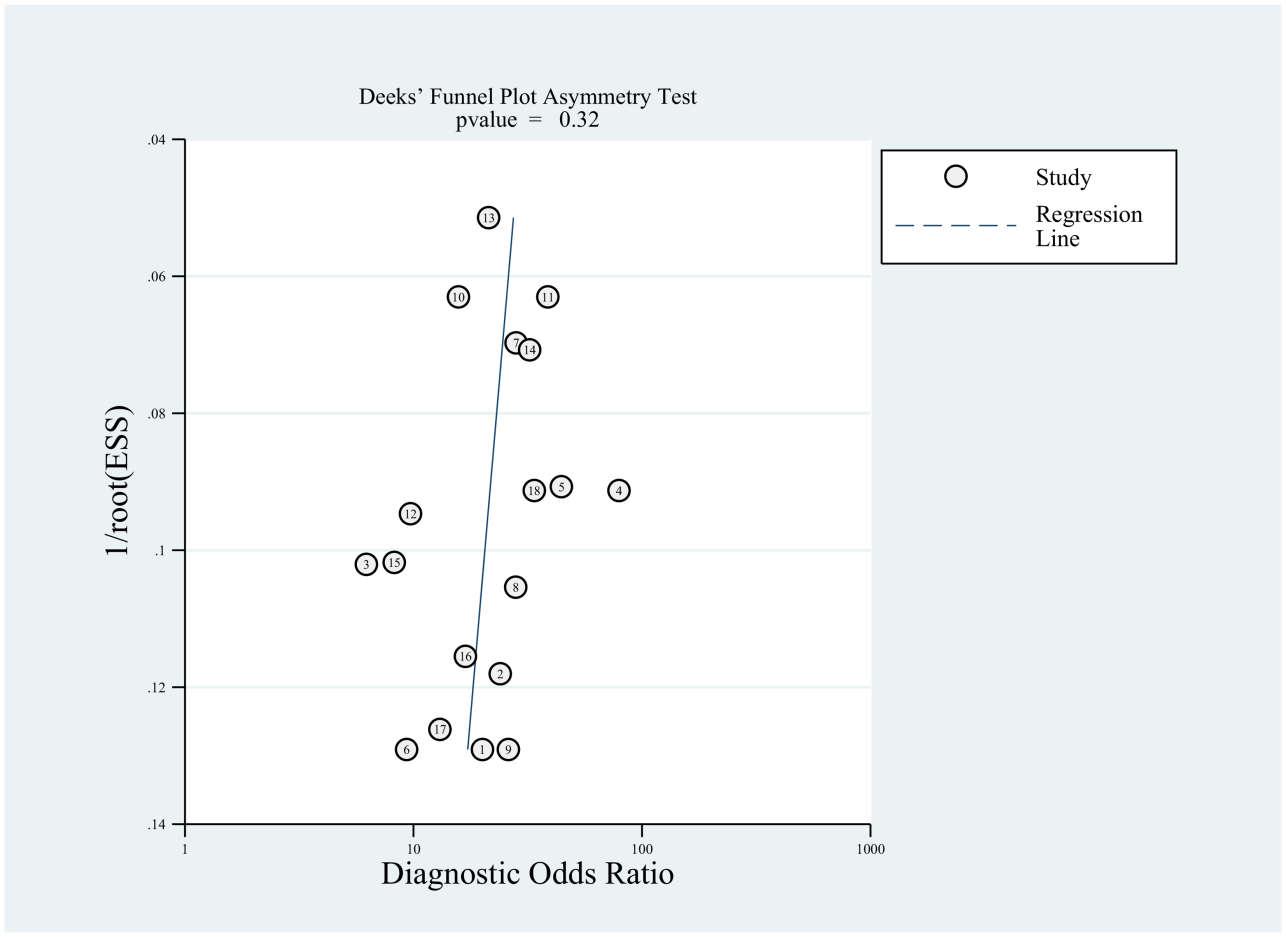
Publication bias analysis of the included studies by Deeks’ funnel plot asymmetric linear regression test.

Sensitivity analyses were executed to examine the reliability of the findings. These analyses demonstrated that the outcomes of the meta-analysis remained largely consistent even when individual studies were removed one by one. This consistency suggested the absence of significant bias in the included studies and confirmed the reliability and stability of the results (Figure 5). Furthermore, Deeks’ funnel plot was employed to assess publication bias, revealing no notable publication bias (*p* = 0.32; Figure 6).

### 3.4 Descriptive analysis

Zhou et al. (11) showed that PBMC miR-29b as a diagnostic for tuberculosis was specific (90.5%), but not sensitive (56%) in children. When compared with healthy controls, PBMC miR-29 was upregulated in the TB cases. Zhang et al. (35) found that the performance values of miR-29c are 72.6, 82.8% and 0.846 for sensitivity, specificity, and AUC value, respectively. Meanwhile, they found that the expression levels of miR-29c in TB serums were higher than those in normal control serums.

Yi et al. (26) detected miR-29a expression in the sputum of patients with pulmonary TB, and their results showed that miR-29a exhibited a sensitivity of 80%, a specificity of 85%, and an AUC value of 0.867 for the diagnosis of TB. Pan et al. (33) detected miR-29a expression in cerebrospinal fluid of children with tuberculosis meningitis. Their results showed that miR-29a expression was considerably elevated in the cerebrospinal fluid of children with tuberculosis meningitis. The sensitivity, specificity, and AUC of miR-29a in cerebrospinal fluid for the diagnosis of tuberculosis meningitis were 81.1, 90.0%, and 0.890, respectively.

## 4 Discussion

In this systematic review and meta-analysis, the diagnostic accuracy of the miR-29 family as a TB biomarker was assessed. This study showed that miR-29 family has high diagnostic efficacy accuracy in detecting TB, and the pooled sensitivity and specificity of miR-29a were 82 and 82%, respectively, which demonstrates that the missed diagnosis and misdiagnosis rates of TB using miR-29 have been as low as 18 and 18%, respectively. Results of our subgroup analysis in sensitivity and specificity also supported the above assumption. These findings were similar to Yao et al.’s study (36). As the sensitivity and specificity were higher than 80%, the diagnostic accuracy of miR-29a for tuberculosis was quite high. In addition, higher than 5 of positive likelihood ratio and lower than 0.3 of negative likelihood ratio are considered good diagnostic significance (37). In our meta-analysis, the positive likelihood ratio was 4.7, indicating that the probability of a miR-29a-positive diagnosis of TB was 4.7-fold higher than that of non-TB controls. DOR is a measure of diagnostic test efficiency that combines sensitivity and specificity; the higher the value of DOR, the better the performance of the discriminatory test (38). In this study, the DOR was 21, indicating that miR-29a is a good marker to distinguish TB from non-TB groups. The SROC curve also suggested that miR-29a had an excellent performance in diagnosing TB; the AUC value reached 0.89, which represents a high over accuracy with high values of sensitivity and specificity. Therefore, miR-29a could be an accurate molecular marker to support the diagnosis of TB. Nevertheless, it cannot be used as the only diagnostic marker of TB as it has a misdiagnosis rate of 18%. In addition, miR-29b and miR-29c also had a good performance in diagnosis of tuberculosis.

Notably, miR-29 exhibited reduced expression in IFN-γ-producing cells such as (natural killer) NK cells and CD4 + and CD8 + T cells following *Mycobacterium tuberculosis* BCG infection in mice (14). Its expression significantly increased in both latent and active TB cases, leading to reduced IFN-γ levels and modifications in signaling pathways (39). Ndzi EN et al. (31) also indicated that miR-29a demonstrated strong performance in distinguishing active pulmonary TB from healthy subjects (AUC = 81.37%) and exhibited good diagnostic performance in distinguishing active pulmonary TB from latent pulmonary TB (AUC = 84.35%). The performance of miR-29a-3p present in blood in distinguishing active TB from healthy control groups suggested that it may be a useful biomarker for TB diagnosis. The presence of this miRNA in readily obtainable blood (plasma) samples suggests its potential utility in diagnosing TB, particularly in children and cases of extrapulmonary TB, as compared to sputum-based methods. The findings also substantiated the aforementioned viewpoints. miR-29a exhibited a sensitivity of 82% and a specificity of 81% for TB diagnosis in the adult population and a sensitivity of 74% and a specificity of 89% for pediatric TB diagnosis. Moreover, this study also indicated that miR-29a, sourced from various specimen types, demonstrated strong performance in effectively distinguishing TB cases from healthy control groups.

miRNA is a single-stranded RNA molecule typically spanning 18 to 25 nucleotides in length. Its primary function is to restrict the activity of target genes during the post-transcriptional phase of gene expression. miRNA is kind of RNA that does not encode proteins. Instead, its final transcripts engage with the mRNA of target genes. miRNA serves to regulate the translation of mRNA, along with other regulatory components, including transcription factors (40). The majority of miRNAs are deemed to be situated within genomic regions classified as non-coding. An estimated 2 to 5% of human genes across the entire genome are believed to encode miRNAs (41). In many cases, Multi-gene transcripts often contain the genetic code for miRNAs. It is estimated that miRNAs possess the ability to regulate more than one-third of human genes, primarily because a single miRNA can target multiple mRNA molecules (42). Furthermore, various studies have demonstrated differences in gene expression profiles of macrophages and NK cells among participants with active TB, latent TB, and healthy controls. As reported by Fu et al. (10) and Sharbati et al. (43), miR-29 was initially identified as an mRNA repressor with a specific target: the HIV-13’UTR region in viral infections. Notably, during systemic infections caused by *Mycobacterium bovis*, miR-29 exhibited upregulation in NK cells, leading to the inhibition of the target gene IFN-γ. The miR-29a-mediated pathway enhanced host resistance to Listeria infection. Conversely, miR-29a demonstrated upregulation in both serum and sputum samples acquired from individuals with active pulmonary TB in comparison to healthy control groups. During *Mycobacterium avium* infection in human macrophages, the expression of miR-29a was also induced. This induction led to the targeting of genes involved in the apoptotic pathway, specifically caspase 7 and 3. Consequently, miR-29a exerted control over alterations in the composition of immune cells and the gene expression of associated target genes under TB infection (44).

miR-29a is the miRNA related to *Mycobacterium tuberculosis* infection (45). *Mycobacterium tuberculosis* infection leads to overexpression of miR-29a in host cells. miR-29a suppresses immune responses to *Mycobacterium tuberculosis* by reducing IFN-γ levels. Apart from its targeting of the 3′ untranslated region of IFN-γ mRNA, miR-29a also facilitates the binding of IFN-γ mRNA to the protein Argonaute 2. This interaction results in the formation of an RNA-silencing complex, which then leads to the post-transcriptional reduction of IFN-γ expression (46). Furthermore, multiple studies have provided evidence that miR-29a also targets genes such as anti-apoptotic B-cell lymphoma 22, myeloid leukemia-1, GTP-bound cell division cycle 42, and the p85 kinase gene, underscoring its involvement in the regulation of apoptotic pathways. In the anti-TB response, overexpression of miR-29a in TB infection hinders macrophages from engulfing tubercle bacilli by suppressing IFN-γ and promoting apoptosis (10).

Despite some promising results, significant heterogeneity differences between studies were observed. Numerous factors encompassing sample type, sample storage and processing, miRNA profiling techniques, validation techniques, and data normalization procedures may bring about potential variations, potentially resulting in inconsistent findings. Because there was a scarcity of available studies for analysis, it was not feasible to conduct additional subgroup analyses on the basis of sample sources or profiling methods. Furthermore, variations in reporting, with some studies including-3p or -5p variants while others did not, may have contributed to a potential reduction in the precision of the analysis. An additional significant limitation pertained to the relatively small sample sizes in each of the included studies. In biomarker discovery studies, the cohort size assumes critical importance and should ideally be representative of the target population. Furthermore, most of the studies were from China, which might lead to the patient selection bias and might represent the clinical characteristics of Chinese patients with tuberculosis. In addition, most of the subjects in the study population were from China and India with the highest burden of TB. miR-29 sensitivity might be lower in low- countries such as European and American countries. As a screening marker for tuberculosis, the cut-off value of miR-29 is supposed to be reduced in these low-burden countries to increase its sensitivity. Moreover, it is essential to diagnose active tuberculosis and latent tuberculosis infection to control the spread of *Mycobacterium tuberculosis* (Mtb), a complex pathogen increasingly resistant to antibiotics. Unfortunately, due to the small amount of available literature concerning miR-29 diagnostic efficacy between latent pulmonary TB and active pulmonary TB, the authors were unable to confirm these findings with literature sources. Nonetheless, despite these acknowledged limitations, a comprehensive pooled analysis of available data from the miR-29 family was carried out to examine the involvement of miR-29 in the field of TB.

Progress in science and technology has opened avenues for the development of diverse innovative miRNA detection platforms. These include Bead Array-based profiling, microfluidic technology, miRNA activity reporting assays, and amplification assays (cycle-mediated isothermal amplification, exponential isothermal amplification, rolling circle amplification). These platforms hold the potential to facilitate the creation of rapid and cost-effective assays for the sensitive diagnosis of TB. Additionally, these platforms allow for the identification of candidate miRNAs that are poised to become the most promising in this context (47). Using the miR-29 family as a diagnostic reagent will aid in developing rapid, cost-effective assays for TB, avoiding the shortcomings of traditional diagnostic tests.

## 5 Conclusion

The meta-analysis conducted in this study has demonstrated that miR-29 exhibits the potential to distinguish active TB patients from healthy control groups. It has high performance in diagnosing both active TB and extrapulmonary TB across both pediatric and adult patient populations. Consequently, the most studied circulating miRNA, especially circulating miR-29a, may be a highly accurate diagnostic tool for tuberculosis. Nevertheless, it is imperative to underscore that further large-scale prospective studies are necessary to validate the findings of this investigation before considering the clinical implementation of miR-29 as a diagnostic marker.

## Data availability statement

The original contributions presented in the study are included in the article/Supplementary material, further inquiries can be directed to the corresponding author.

## Author contributions

JH: Conceptualization, Data curation, Formal analysis, Funding acquisition, Investigation, Methodology, Project administration, Resources, Software, Supervision, Validation, Visualization, Writing – original draft, Writing – review & editing. JX: Methodology, Software, Writing – original draft. YH: Formal analysis, Investigation, Methodology, Software, Writing – original draft.

## Footnotes

^1^ http://www.crd.york.ac.uk/PROSPERO
