# Supplementary material for: miR-29 as diagnostic biomarkers for tuberculosis: a systematic review and meta-analysis
Source: Front Public Health. 2024 May 14;12:1384510. doi: 10.3389/fpubh.2024.1384510 (PMC11130415; doi:10.3389/fpubh.2024.1384510)
Supplement: Supplementary file 1 [file Table_1.DOCX]

**Supplemental Material**

Supplementary Table 1. Search strategies in each database

| **Database** | **Search strategies** | **No. of studies** |
| --- | --- | --- |
| **PubMed** | #1 ("MIRNA29a microRNA, human" [Supplementary Concept]) OR (miR-29[Title/Abstract] OR miRNA-29[Title/Abstract] OR microRNA-29[Title/Abstract] OR small RNA-29[Title/Abstract])---1,744  #2 ("Diagnosis"[Mesh]) OR (Diagnosis[Title/Abstract] OR prognosis[Title/Abstract] OR Diagnosis[Title/Abstract] OR diagnostic[Title/Abstract] OR detect[Title/Abstract] OR predict[Title/Abstract] OR prognostic[Title/Abstract] OR screen[Title/Abstract] OR distinguish[Title/Abstract] OR differentiate[Title/Abstract] OR discriminate[Title/Abstract]) ---11,526,015  #3 (("Tuberculosis"[Mesh]) OR ("Mycobacterium tuberculosis"[Mesh])) OR (Tuberculosis [Title/Abstract] OR Mycobacterium tuberculosis [Title/Abstract] OR TB[Title/Abstract] OR MTB[Title/Abstract] OR "M. Tuberculosis"[Title/Abstract]) ---302,373  #4 #1 AND #2 AND #3 ---27 | 27 |
| **Web of**  **Science** | #1 ((TS=(miR-29)) OR TI= (miR-29 OR miRNA-29 OR microRNA-29 OR small RNA-29)) OR AB= (miR-29 OR miRNA-29 OR microRNA-29 OR small RNA-29) ---1,392  #2 ((TS=(Diagnosis)) OR TI= (Diagnosis OR prognosis OR Diagnosis OR diagnostic OR detect OR predict OR prognostic OR screen OR distinguish OR differentiate OR discriminate)) OR AB= (Diagnosis OR prognosis OR Diagnosis OR diagnostic OR detect OR predict OR prognostic OR screen OR distinguish OR differentiate OR discriminate) ---6,510,090  #3 ((TS=(Tuberculosis OR Mycobacterium tuberculosis)) OR TI=(Tuberculosis OR Mycobacterium tuberculosis OR TB OR MTB OR “M. Tuberculosis")) OR AB=(Tuberculosis OR Mycobacterium tuberculosis OR TB OR MTB OR “M. Tuberculosis")---151,001  #1 AND #2 AND #3---37 | 37 |
| **Embase** | #1 'mir 29'/exp OR 'mir 29': ti, ab, kw OR 'mirna 29': ti, ab, kw OR 'microrna 29': ti, ab, kw OR 'small rna-29': ti, ab, kw---2,910  #2 'diagnosis'/exp OR prognosis: ti, ab, kw OR diagnosis: ti, ab, kw OR diagnostic: ti, ab, kw OR detect: ti, ab, kw OR predict: ti, ab, kw OR prognostic: ti, ab, kw OR screen: ti, ab, kw OR distinguish: ti, ab, kw OR differentiate: ti, ab, kw OR discriminate: ti, ab, kw--- 11,286,190  #3 'tuberculosis'/exp OR 'mycobacterium tuberculosis'/exp OR tuberculosis: ti, ab, kw OR 'mycobacterium tuberculosis': ti, ab, kw OR tb: ti, ab, kw OR mtb: ti, ab, kw OR 'm. tuberculosis':ti, ab, kw --- 396,836  #4 #1 AND #2 AND #3---35 | 35 |
| **Cochrane Library** | #1 (miR-29 OR miRNA-29 OR microRNA-29 OR small RNA-29):ti, ab, kw---446,376  #2 (MeSH descriptor: [Diagnosis] explode all trees) OR ((Diagnosis OR prognosis OR Diagnosis OR diagnostic OR detect OR predict OR prognostic OR screen OR distinguish OR differentiate OR discriminate):ti, ab, kw)) ---784,891  #3 (MeSH descriptor: [Tuberculosis] explode all trees) OR ((Tuberculosis OR Mycobacterium tuberculosis OR TB OR MTB): ti, ab, kw)---9,377  #4 #1 AND #2 AND #3---5 | 5 |
| **CNKI** | #1 ((SU=miR-29) OR (TKA=miR-29 OR miRNA-29 OR microRNA-29 OR small RNA-29)) AND ((SU=Diagnosis) OR (TKA=Diagnosis OR prognosis OR Diagnosis OR diagnostic OR detect OR predict OR prognostic OR screen OR distinguish OR differentiate OR discriminate)) AND ((SU=Tuberculosis OR Mycobacterium tuberculosis) OR (TKA=Tuberculosis OR Mycobacterium tuberculosis OR TB OR MTB OR “M. Tuberculosis"))---28 | 28 |
